# Aberrant CpG‐methylation affects genes expression predicting survival in lung adenocarcinoma

**DOI:** 10.1002/cam4.1834

**Published:** 2018-10-23

**Authors:** Wei He, Dandan Ju, Zhijun Jie, Ai Zhang, Xin Xing, Qin Yang

**Affiliations:** ^1^ Department of Respiratory Medicine The Fifth People’s Hospital of Shanghai, Fudan University Shanghai China; ^2^ Obstetrics and Gynecology Hospital of Fudan University Shanghai China; ^3^ The People's Hospital of Shanghai Pudong District Shanghai China; ^4^ Department of Obstetrics and Gynecology Fengxian Hospital Shanghai China; ^5^ State Key Laboratory of Oncogenes and Related Genes Shanghai Cancer Institute, Ren Ji Hospital, School of Medicine, Shanghai Jiao Tong University Shanghai China

**Keywords:** lung adenocarcinoma, methylation, RNAseq

## Abstract

Lung adenocarcinoma (LUAD) is a common diagnosed disease with high‐mortality rate, and its prognostic implications are under discovered. DNA methylation aberrations are not only an important event for dysregulation of gene expression during tumorigenesis but also a revolution in epigenetics by identifying key prognostic biomarkers for multiple cancers. In this study, we analyzed methylation status of 485 578 CpG sites and RNA‐seq transcriptomes of 20 532 genes for 1095 LUAD samples in TCGA database. The association between DNA methylation and the prognostic value of the corresponding gene expression was identified as well. In total, ten aberrantly methylated and dysregulated genes (AURKA, BLK, CNTN2, HMGA1, PTTG1, TNS4, DAPK2, MFSD2A, THSD1, and WNT7A) were highlighted which were significantly correlated with overall survival of 492 LUAD patients, which were all reported as tumor‐associated genes in other various cancers and worthy of further investigated and might be used as therapeutic targets for LUAD. Together, methylation aberrances regulate gene expression level during tumorigenesis and influence prognosis of LUAD patients. Integrating knowledge of epigenetics and expression of genes can be useful for an in‐depth understanding of cancer mechanism and for the eventual purpose of precisely prognostic and therapeutic target verification.

## 1 INTRODUCTION

Lung cancer (LC) is a common diagnosed disease in both women and men around the world, which has high‐mortality rate and is responsible for approximately 1.5 million deaths every year.^1^ According to the histological heterogeneity, LC is divided into two primary subtypes, non‐small cell lung cancer (NSCLC) and small cell lung cancer (SCLC), representing for 85% and 15% of all LC, respectively.^2^ NSCLC can be further subdivided into lung adenocarcinoma (LUAD), lung squamous cell carcinoma (LUSC) and lung large cell carcinomas (LULC). Among them, LUAD has increased up to 50% and becomes the biggest subgroup of LC since early 2010s.^3^ Up to date, the overall 5‐year survival rate of LUAD patients is approximately 20%; however, it rises to 55% in the cases diagnosed with localized lung cancer. With the rapidly increasing morbidity and severe metastasis‐associated mortality, it is crucial to clarify the molecular mechanisms and oncogenomic aberrations, which characterize the occurrence and metastatic process of LUAD.

It is well‐established that genetic dysregulation is the key part of cancer etiology.^4^ In addition, new emerging evidences have demonstrated the combined effect of both genetic and concomitant epigenetic change must be considered during oncogenesis.^5^ Oncogenomic aberrations are no longer to be taken not only as a genetic disorder exclusively, but also as epigenetic alterations. DNA methylation, an important form of epigenetic modification, has significant functions on gene expression, genomic stability, and modification.^6^ Hypermethylation or hypomethylation of DNA was observed in variety of tumors but not in various normal tissues,^7^ which indicated that methylation aberration might be treated as a hallmark of a wide variety of cancers.

The advent of deep RNA‐Seq approach and wide DNA methylation arrays has significantly contributed to explore the interactive relationship between gene expression and methylation during tissue development and carcinogenesis. Xie et al^8^ investigated the significant importance of DNA methylation on modulating gene expression monitored by RNA‐Seq analysis during human heart, kidney and liver development. An integrative analysis of DNA methylation and mRNA expression performed by Kim et al^9^ pointed out the key function of epigenetic alteration on human malignant mesothelioma cell heterogeneity. However, the methylation state of LUAD‐specific‐associated genes is still under investigation. In the present study, we analyzed large‐scale DNA methylation level and RNA‐seq transcriptomes of LUAD samples from 1095 cases in TCGA database. Together with the survival analysis of 492 LUAD patients, 10 potential diagnostic and prognostic biomarkers of LUAD were pointed out which were worthy of further investigated and might be used as therapeutic targets for LUAD.

## 2 METHODS

### 2.1 Patients and data processing

In this study, 1095 LUAD cases in total were downloaded from TCGA data portal (https://cancergenome.nih.gov/, level 3， normalized gene expression data [RSEM] and HumanMethylation450 data) accessed on 20171206. Of them, 636 LUAD patients had whole genomic DNA methylation data of 485 578 CpG sites, which was profiled by using Illumina Infinium HumanMethylation450 BeadChips assay. And 576 LUAD sufferers had transcriptomic data of 20 532 genes, which was analyzed using Illumina HiSeq_RNASeq V2 platform. A total of 492 of these 576 LUAD suffers had recorded clinical annotation data and involved in further Kaplan‐Meier survival analysis.

**Figure 1:**
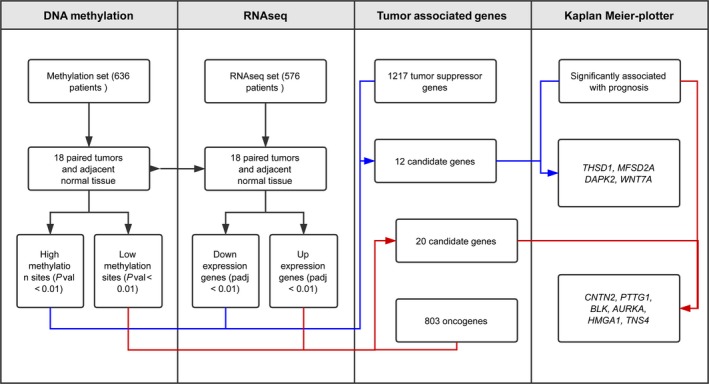
Flowchart representing the design of study. First, the methylation status of CpG sites and the mRNA expression level of transcripts in 18 paired LUAD and adjacent non‐LUAD tissues from 1095 cases of LUAD were compared. Then, 32 candidate genes were narrowed down by intersection of aberrant methylated genes and abnormal expressed transcripts, as well as tumor‐associated genes. Finally, together with survival rate analysis of 492 LUAD patients, we found out 10 prognostic‐related genes which were differentially expressed in LUAD tissues due to their aberrant methylation of CpG sites

To precisely discover the differential methylation CpG sites and transcripts, we only selected the cases which had data for both LUAD tumor and adjacent non‐LUAD normal tissues. Finally, 18‐paired cases were co‐existed in 29 coupled (tumor and adjacent tissue) methylation data and 57 coupled RNA‐seq data, which was used in the followed methylation and expression analysis (Figure 1).

### 2.2 DNA methylation analysis

We computed the difference at the probe level between the tumor and normal groups in LUAD by using R Bioconductor minfi package with version 1.24.0.^10^ According to the annotations provided by Illumina for the HumanMethylation450 platform (IlluminaHumanMethylation450kanno.ilmn12.hg19), only probes mapped uniquely to the human reference genome (hg19) were kept for analysis in this study. The methylated genes, which have significant differences termed biologically meaningful for an FDR q‐value below 0.01, were further categorized into hypermethylation and hypomethylation subgroups, according to their mean value for the 18 LUAD patients higher than 0.7 or less than 0.3, respectively. The Methylation_450 value of hypermethylated and hypomethylated genes was used for heatmap figure with one additional scale normalization step, which subtracted the mean value from Methylation_450 then dividing by the standard deviation of Methylation_450 value.

### 2.3 Differential expression analysis of transcripts

All the normalized gene expression RSEM data was transformed into RNA‐seq read counts by tximport method.^11^ Next, an existing method called DESeq2 was used to determine the transcripts that were differentially expressed between LUAD and adjacent normal tissues. We prepared an input matrix of RNA‐seq read counts where rows were transcripts and columns were paired tumor‐normal samples. The transcripts with absolute log fold changes (logFC) greater than 2 and FDR‐corrected values less than 0.01 were termed to be differentially expressed. Similar to making the heatmap of methylation analysis, the heatmap of differentially expressed genes also utilized normalized scale value, which removing the mean value from normTransform (log2) DESeq2 followed with dividing by the standard deviation of normTransform (log2) DESeq2 value.^12^

### 2.4 Estimation of survival value

Kaplan‐Meier curves, with *P*‐values calculated via log‐rank test was used to represent the survival distributions between “high” and “low” expression groups (defined by median value of each gene expression). Two‐sided *P* values, which calculated by R survival package^13^, ^14^ lower or equal than 0.05 were considered statistically significant.

## 3 RESULTS

### 3.1 Identifying CpG based on genomewide profiling

**Figure 2:**
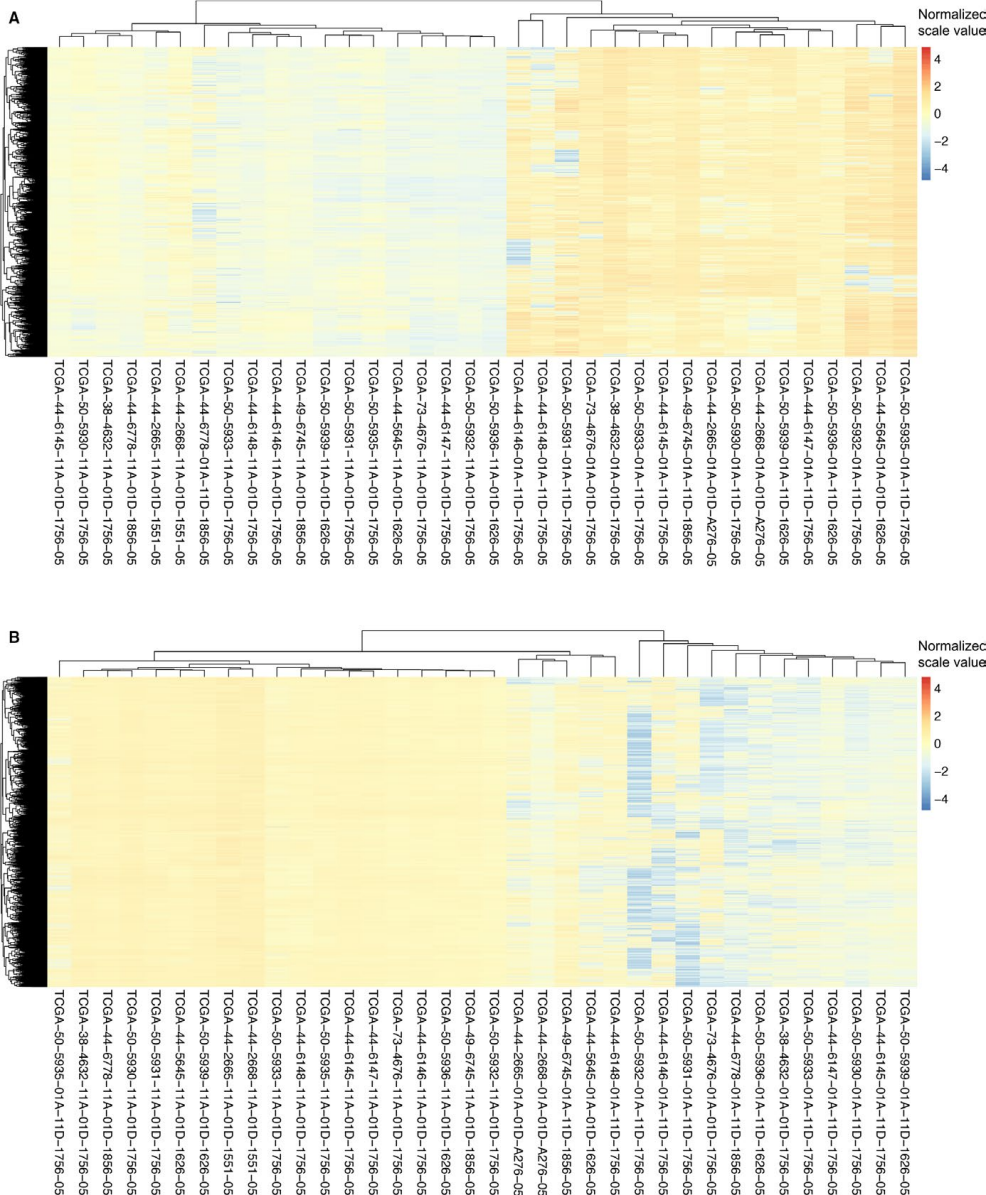
Aberrant methylation of genes in LUAD. A, Heatmap of 2087 hypermethylated genes in tumor tissue compared to adjacent normal tissue of 18 paired LUAD patients. B, Heatmap of 5416 hypomethylated genes in LUAD tissues compared to matched adjacent normal tissue

Given that methylation of CpG dinucleotides represents more than 98% of DNA methylation in mammalian somatic cells,^15^ we focused on comparison of CpG sites methylation across the genome of LUAD tumors and normal tissue. From 636 patients in TCGA, a total of 18 pairs of LUAD tissues and matched healthy tissues were picked up and involved in present DNA methylation study (Figure 1). The methylation distribution for all paired patients abides by a bimodal distribution, with peaks around 0 (un‐methylated) and 1 (methylated). The heat maps showed that hypermethylation of 2087 genes (Figure 2A) and hypomethylation of 5416 genes (Figure 2B) were identified for 18 pairs of tumor and adjacent normal tissue, based on the genomewide analysis of CpG methylation (*P*‐value <0.01).

### 3.2 Differentially expressed mRNAs in LUAD

**Figure 3:**
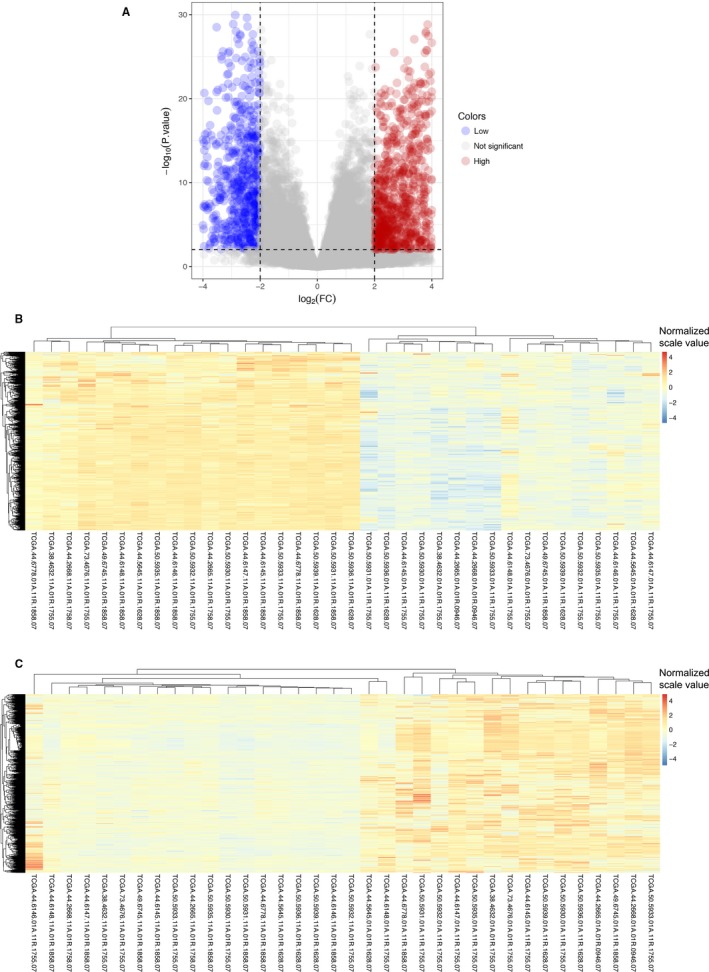
Differentially expressed mRNA in LUAD. A, Volcano plot displayed the situation of dysregulated mRNA for LUAD tumor and non‐tumor tissues. A total of 1829 mRNAs showed an absolute value of log fold change greater than 2 and FDR‐corrected values less than 0.01 (Blue and red dots). B, Heatmap showed 696 down‐regulated mRNA in tumor tissue compared to adjacent normal tissue of 18 paired LUAD patients. C, Heatmap showed 1133 up‐regulated mRNA in tumor tissue compared to adjacent normal tissue of 18 paired LUAD patients

RNA‐seq data of the same 18 matched LUAD tumors and adjacent normal tissues were involved in the differential expression analysis. According to the cutoff criteria (|logFC|≥2, Padj <0.01), 1829 mRNAs were differentially expressed between LUAD and matched healthy tissues (Figures 1 and 3A). Among them, 696 genes were down‐regulated and 1133 genes were up‐regulated. The results of expression analysis were presented as heat maps to demonstrate that the down‐regulated (Figure 3B) and up‐regulated (Figure 3C) genes in all 18 pair of patients.

### 3.3 Selection of candidate genes for LUAD prognostic biomarkers

**Figure 4:**
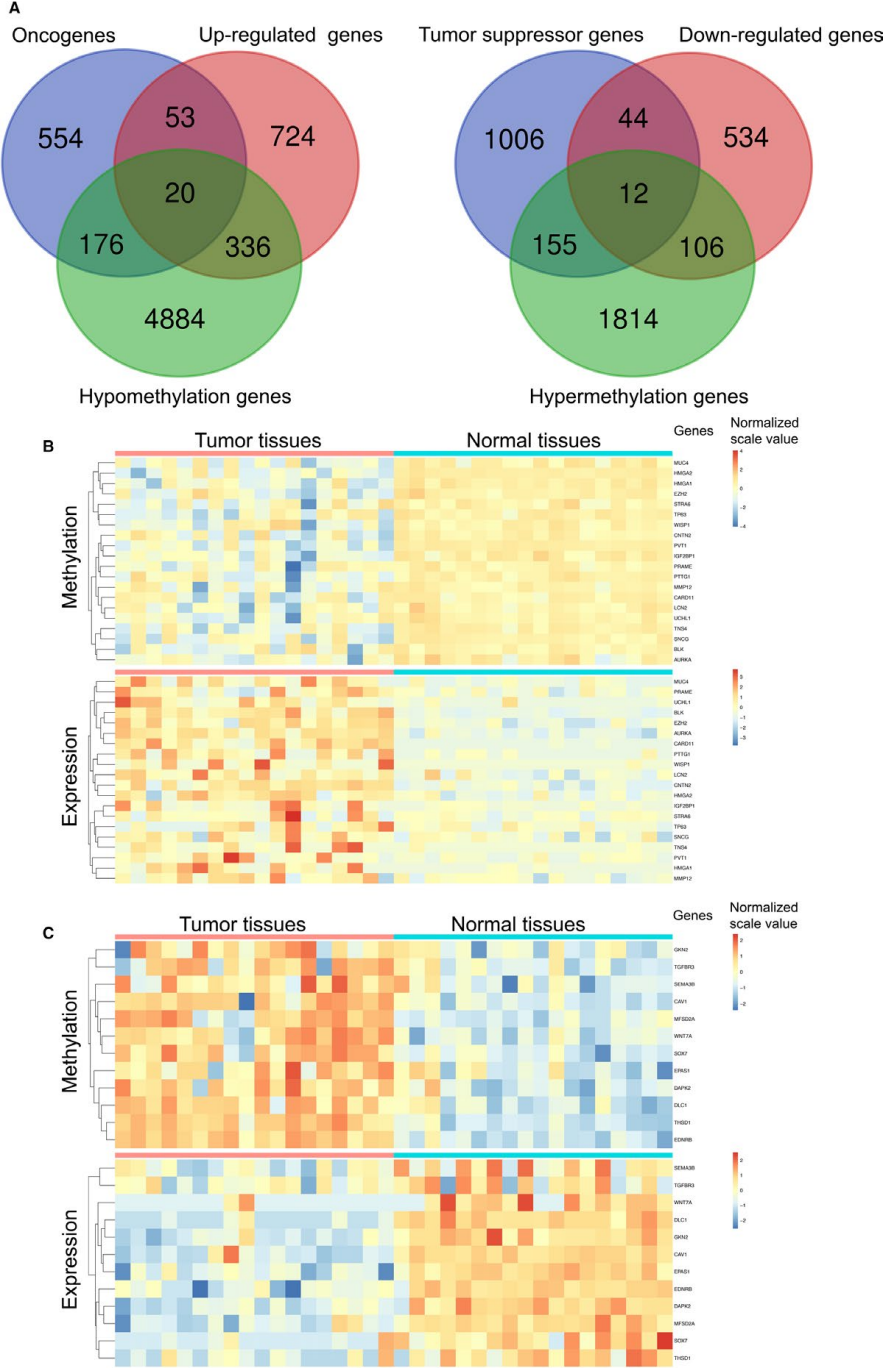
32 known tumor‐associated genes were identified as methylation‐based abnormal expression in LUAD. A, Venn diagrams represented 20 hypomethylation‐up‐regulated‐oncogenes and 12 hypermethylation‐down‐regulated‐tumor suppressor genes in tumor tissue compared to adjacent normal tissue of 18 paired LUAD patients. B, Heatmaps of 20 hypomethylation‐up‐regulated‐oncogenes in LUAD tissues compared to matched adjacent normal tissue. C, Heatmaps of 12 hypermethylation‐down‐regulated‐tumor suppressor genes in LUAD tissues compared to matched adjacent normal tissue

Up to date, 803 oncogenes and 1217 tumor suppressor genes were currently defined by human cancer studies (https://ongene.bioinfo-minzhao.org/ and https://bioinfo.uth.edu/TSGene). Together with aberrant methylation sites and differential expression genes as well as the known oncogenes or tumor suppressor genes, 32 genes were identified and classified into two groups, 20/32 genes in group I (hypomethylation‐up‐regulated‐oncogenes), and 12/32 genes in group II (hypermethylation‐down‐regulated‐tumor suppressor genes) (Figures 1 and 4A). The heatmaps of all 32 genes in paired tumor and normal tissues indicated that the negative association between aberrant situation of DNA methylation and mRNA expression level (Figure 4B and C).

**Figure 5:**
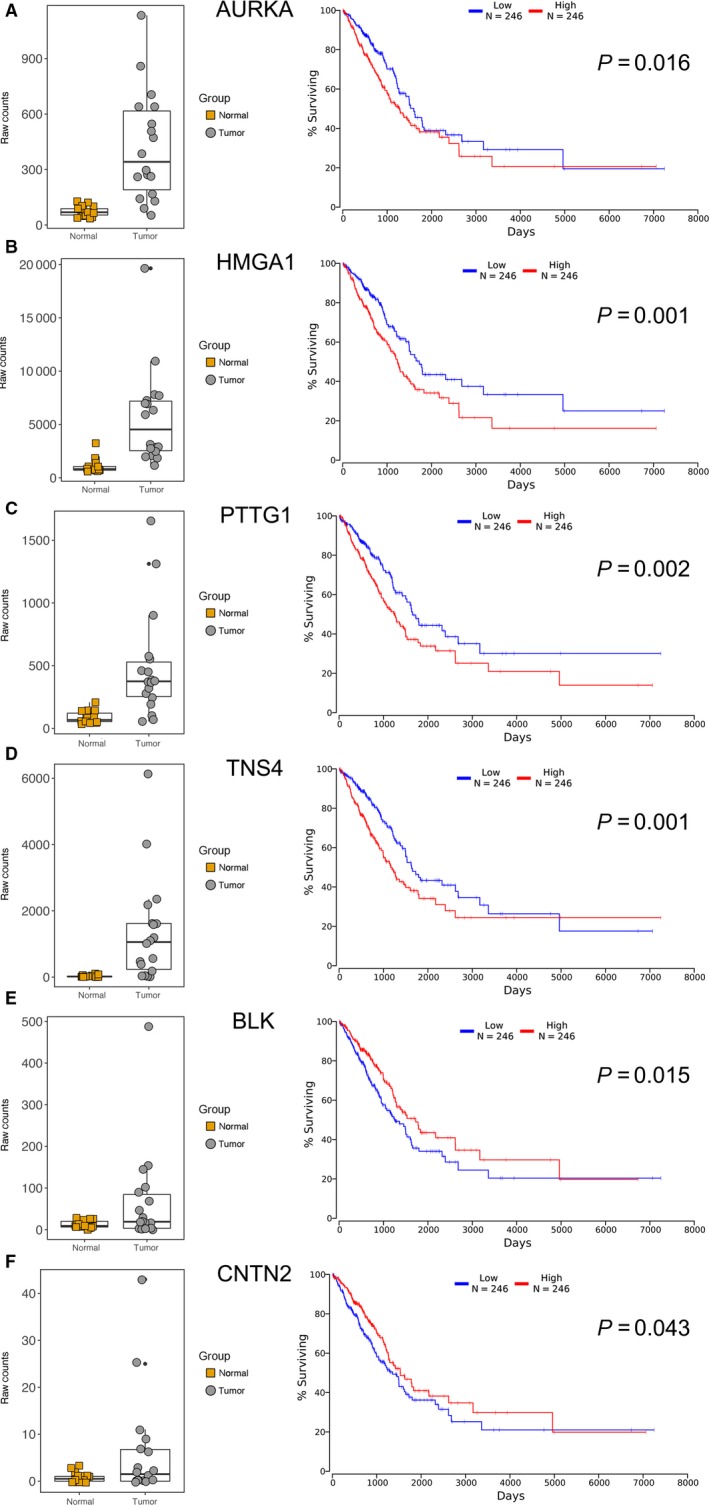
Differential expression and prognostic signature of six candidate oncogenes in LUAD patients. A‐F, Left panels indicated the overexpression of six candidates in LUAD tissues compared to matched adjacent normal tissue. Right panels showed Kaplan‐Meier curves of 492 LUAD patients, who were separated into high‐expressed and low‐expressed groups using a cutoff of median value of different genes. All the values were reached significance (*P* < 0.05)

**Figure 6:**
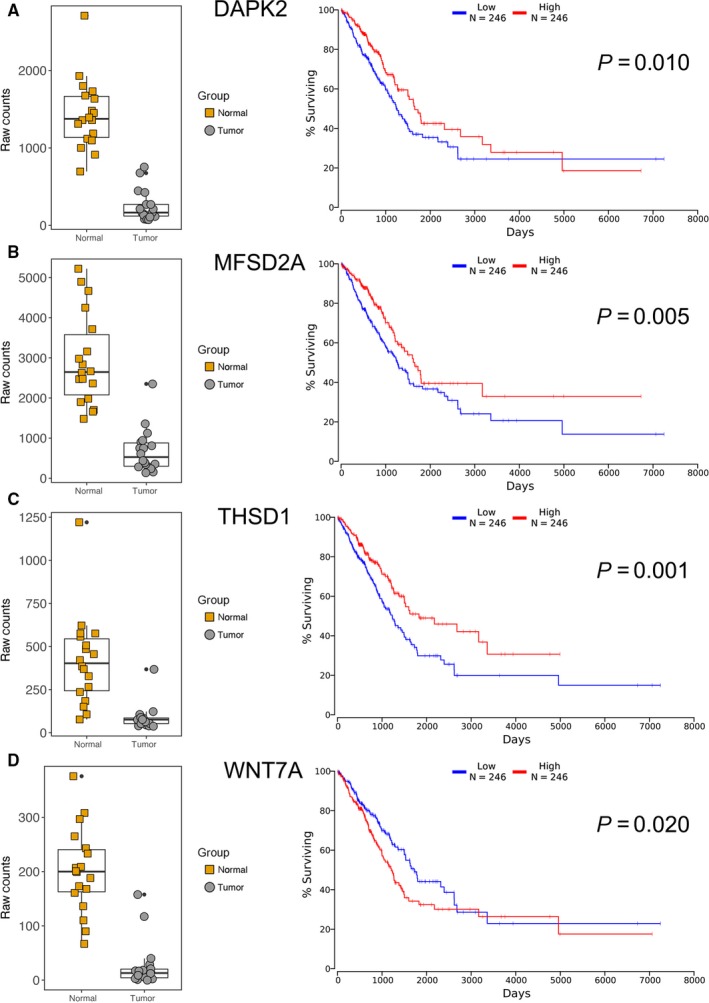
Differential expression and prognostic signature of four candidate tumor suppressor genes in LUAD patients. A‐D, Left panels indicated the down‐regulated of four candidates in LUAD tissues compared to matched adjacent normal tissue. Right panels showed Kaplan‐Meier curves of 492 LUAD patients, who were separated into high‐expressed and low‐expressed groups using a cutoff of median value of different genes. All the values were reached significance (*P* < 0.05)

Furthermore, Kaplan‐Meier plotter analysis was performed on these 32 genes for verifying the genes, which correlated with prognosis of LUAD. A total of 492 LUAD patients were stratify into two groups according to the median expression level were involved in this study. A univariate Cox proportional hazards regression analysis showed six genes (AURKA, BLK, CNTN2, HMGA1, PTTG1, and TNS4, Figure 5) from group I, four genes (DAPK2, MFSD2A, THSD1 and WNT7A, Figure 6) from group II were significantly associated with the survival of 492 LUAD patients. Surprisingly, among these genes, BLK, CNTN2 (Figure 5E and F) and WNT7A (Figure 6D) displayed reversed relationship between their expression level and survival rate of LUAD patients. Nevertheless, all these 10 candidate genes should be considered as notable prognostic biomarkers and promising therapeutic targets in further experimental research.

## 4 DISCUSSION

With the high‐mortality rate, LUAD is responsible for the majority of tumor‐related deaths. However, increasing numbers of somatic mutations and genomic dysregulations have been discovered in LUAD, which makes the identification of the key driver gene alterations challenging. A number of studies have shown that DNA methylation is the second “motivation” of carcinogenesis after gene mutation and has become an important marker for early tumor diagnosis.^16^, ^17^

Compared to gene mutations, abnormalities of DNA methylation are more common in tumor genomes and reversible according to various factors such as genetic background, age, environment, diet, and behavior. In addition, it can dynamically influence the gene status and eventually lead to tumorigenesis.^18^ With the advantages of new‐generation sequencing technologies, the methylation status has been detected on the whole genome level. In this study, we identified 2087 hypermethylated genes and 5416 hypomethylated genes from 18 pairs of LUAD and control tissues.

The occurrence of tumors is a complex process regulated by genetic, environmental, and epigenetic factors, which results in significant individual differences of cancer patients. In clinical practice, individualized diagnosis is an important prerequisite for appropriate treatment. Epigenetic markers based on DNA methylation and mRNA expression is indispensable. Toyooka et al^19^ found DNA methylation was ubiquitous in all stages of lung cancer development and negatively regulated the expression of oncogenes and tumor suppressor genes. Therefore, the combination of aberrant DNA methylation and abnormal mRNA expression as well as cancer‐associated gene expression is of great significance for selection of diagnostic and prognostic molecular marker of LUAD. As the results, we found 20 oncogenes and 12 tumor suppressor genes were differentially expressed caused by abnormal DNA methylation in LUAD.

To further explore the correlation between expression of these 32 genes and survival rate of LUAD patients, we evaluated the prognostic values by univariable Cox regression analysis. Our study verified 10 significant prognostic genes at the epigenetic and transcriptomic levels. Among these 10 candidates, six have been reported as Lung cancer‐related genes. It has been discovered AURKA was highly expressed in LUAD and played important roles in the cell cycle and apoptosis of human LUAD cells.^20^ Chen et al^21^ unraveled HMGA1, a NF‐κB signaling related factor, can be regulated by miR‐26 and associated with prognosis of LUAD patients. PTTG1, which regulates TGFβ1/SMAD3 signaling pathway, has been described a potential immunotherapeutic target for development and metastasis of LUAD.^22^ As lung tumor suppressor genes, MFSD2A could inhibit cell cycle and matrix attachment of lung cancer cells,^23^ DAPK2 was found to induce oxidative stress in A549 cells by regulation of mitochondrial function,^24^ and overexpression of THSD1 could significantly reduce the colony‐forming ability of A549 cells.^25^ However, none of these six genes was studied on their DNA methylation level in LUAD. The aberrant DNA methylation, which was indicated in present study, may explain their abnormal expression in LUAD and indicate a novel strategy for renovation their expression to normal level.

TNS4 is was speculated as an oncogene in digestive tract cancers via direct interaction with phosphorylated MET,^26^ but the potential oncogenic activity of TNS4 in LUAD was firstly suggested in present study. In addition, there were three genes, BLK, CNTN2, and WNT7A displayed contradictory association of their expression and survival rate of 492 patients in our study. A variety of genes were expressed conversely in various tumors, even not unaltered during the development of individual cancer. Yan et al^27^ revealed the protein level of CNTN2 was higher in high‐grade glioma cells and tissues and lower in low‐grade glioma cells and tissues. Also, highly expressed genes in tumor tissue may play anti‐tumor functions through activation immune cells or pathways. Very recently, one study discovered overexpressed SPON2 in hepatocellular carcinoma could promote macrophages recruitment and prevent metastasis of hepatocellular carcinoma cells.^28^ Strikingly, WNT7A was down regulated, via promoter methylation, in many NSCLC cell lines and tissues,^29^ which in accordance with the results of DNA methylation and mRNA expression analysis. However, higher expression level of WNT7A associated with worse prognosis was unexpected and needed to verify by further prognosis analysis or biological experiments, which may discover a new mechanism of how WNT7A affect the progression and prognosis of LUAD.

In present study, the DNA methylation and gene expression status between LUAD and normal tissues was identified for the first time. Together with the survival rate analysis, 10 candidates were highlighted. Although the exact functions of these genes are still unknown. The majority of them could be treated as useful and practical biomarkers to improve prognostic value and survival prediction of LUAD, as well as novel applications for appropriate clinical adjuvant testing.

However, there are some limitations may cause few potential genes undiscovered in 803 known oncogenes. In the Tables S1 and S2 listed additional 13 genes’ expression were nicely correlated with the prognosis of 492 LUAD patients (*P* < 0.001). And most of them had already reported as potential oncogenes, which may contribute the development of LUAD or LUSC. The first limitation is the research literatures about some potential oncogenes were published later than 25th December 2015, which is the deadline of 803 known oncogene collected from the systematic search in PubMed. For example, ARNTL2 could enable LUAD self‐sufficient metastasis,^30^ PRC1 via Wnt/β‐catenin signaling pathway to contribute tumorigenesis of LUAD^31^ and GJB3 was overexpressed in LUSC tumors,^32^ as well as ceRNA FAM83A‐AS1 was reported as part of a possible competitive endogenous RNA network of LUSC,^33^ which all published after that deadline. The second limitation is the keywords, that oncogene or oncogenic or oncoprotein or proto‐oncogene which used in the searching method of 803 known oncogenes study, were not existing in the title or abstract of previous publications. SERPINB5, SLC2A1, MS4A1, SPRR1B, and GJB2 were all dysregulated in LUSC or LUAD and may treat as diagnosis biomarkers or molecular targets of initiation and progression of lung carcinogenesis.^34^, ^35^, ^36^, ^37^, ^38^ The last limitation is low quality of genes were not assigned in 803 known oncogenes study, which may cause some potential oncogenes missed. Such as PFKP, which has already identified as a lung cancer oncogene based on its SNP and mRNA expression profile data.^39^ However, among these ten genes described above, only SERPINB5 was reported its abnormal expression in lung cancer due to aberrant DNA methylation and none of them were studied the correlation between their expression level and prognosis of LUAD patients.

Beyond that, although the expression level of three other genes, NUP62CL, NUP210L, and DPEP2, quite significantly correlated with the survival of 492 LUAD patients, no literatures were found in PubMed to demonstrate the situation of these three genes in any lung cancers, which may point out a novel connection between nucleoporins and lung cancers. Nevertheless, the potential function and meaning all these genes on LUAD need to be verified by further experimental studies.

## CONFLICT OF INTEREST

There is no conflict of interests or financial ties to disclose from any authors.

## References

[1] Siegel RL , Miller KD , Jemal A . Cancer statistics, 2015. CA Cancer J Clin. 2015;65(1):5‐29. 25559415 10.3322/caac.21254

[2] DeSantis CE , Lin CC , Mariotto AB , et al. Cancer treatment and survivorship statistics, 2014. CA Cancer J Clin. 2014;64(4):252‐271. 24890451 10.3322/caac.21235

[3] Meza R , Meernik C , Jeon J , Cote ML . Lung cancer incidence trends by gender, race and histology in the United States, 1973–2010. PLoS ONE. 2015;10(3):e0121323. 25822850 10.1371/journal.pone.0121323 PMC4379166

[4] Hahn WC , Weinberg RA . Rules for making human tumor cells. N Engl J Med. 2002;347(20):1593‐1603. 12432047 10.1056/NEJMra021902

[5] Sadikovic B , Al‐Romaih K , Squire J , et al. Cause and consequences of genetic and epigenetic alterations in human cancer. Curr Genomics. 2008;9(6):394‐408. 19506729 10.2174/138920208785699580 PMC2691666

[6] Jones PA . Functions of DNA methylation: islands, start sites, gene bodies and beyond. Nat Rev Genet. 2012;13(7):484‐492. 22641018 10.1038/nrg3230

[7] Ehrlich M . DNA hypomethylation in cancer cells. Epigenomics. 2009;1(2):239‐259. 20495664 10.2217/epi.09.33 PMC2873040

[8] Xie L , Weichel B , Ohm J , Zhang K . An integrative analysis of DNA methylation and RNA‐Seq data for human heart, kidney and liver. BMC Syst Biol. 2011;5(suppl 3):S4. 10.1186/1752-0509-5-S3-S4 PMC3287572 22784623

[9] Kim MC , Kim NY , Seo YR , Kim Y . An integrated analysis of the genome‐wide profiles of DNA methylation and mRNA expression defining the side population of a human malignant mesothelioma cell line. J Cancer. 2016;7(12):1668‐1679. 27698904 10.7150/jca.15423 PMC5039388

[10] Aryee MJ , Jaffe AE , Corrada‐Bravo H , et al. Minfi: a flexible and comprehensive Bioconductor package for the analysis of Infinium DNA methylation microarrays. Bioinformatics. 2014;30(10):1363‐1369. 24478339 10.1093/bioinformatics/btu049 PMC4016708

[11] Soneson C , Love MI , Robinson MD . Differential analyses for RNA‐seq: transcript‐level estimates improve gene‐level inferences. F1000Res. 2015;4:1521. 26925227 10.12688/f1000research.7563.1 PMC4712774

[12] Love MI , Huber W , Anders S . Moderated estimation of fold change and dispersion for RNA‐seq data with DESeq2. Genome Biol. 2014;15(12):550. 25516281 10.1186/s13059-014-0550-8 PMC4302049

[13] Therneau T. A Package for Survival Analysis in S. version 2.38, 2015 https://CRAN.R-project.org/package=survival.

[14] Therneau TM , Grambsch PM . Modeling Survival Data: Extending the Cox Model. New York, NY: Springer; 2000.

[15] Jin B , Li Y , Robertson KD . DNA methylation: superior or subordinate in the epigenetic hierarchy? Genes Cancer. 2011;2(6):607‐617. 21941617 10.1177/1947601910393957 PMC3174260

[16] Paluszczak J , Baer‐Dubowska W . Epigenetic diagnostics of cancer – the application of DNA methylation markers. J Appl Genet. 2006;47(4):365‐375. 17132902 10.1007/BF03194647

[17] Suzuki H , Maruyama R , Yamamoto E , et al. DNA methylation and microRNA dysregulation in cancer. Mol Oncol. 2012;6(6):567‐578. 22902148 10.1016/j.molonc.2012.07.007 PMC5528344

[18] Esteller M . Cancer epigenomics: DNA methylomes and histone‐modification maps. Nat Rev Genet. 2007;8(4):286‐298. 17339880 10.1038/nrg2005

[19] Toyooka S , Gazdar AF . Methylation profiling of lung cancer: a decade of progress. Mol Cancer Ther. 2011;10(11):2020. 22072805 10.1158/1535-7163.MCT-11-0768 PMC4522280

[20] Zhong N , Shi S , Wang H , et al. Silencing aurora – a with siRNA inhibits cell proliferation in human lung adenocarcinoma cells. Int J Oncol. 2016;49(3):1028‐1038. 27571708 10.3892/ijo.2016.3605

[21] Chen C , Chang JT , Ho YF , et al. MiR‐26 down‐regulates TNF‐alpha/NF‐kappaB signalling and IL‐6 expression by silencing HMGA1 and MALT1. Nucleic Acids Res. 2016;44(8):3772‐3787. 27025651 10.1093/nar/gkw205 PMC4856999

[22] Li WH , Chang L , Xia YX , et al. Knockdown of PTTG1 inhibits the growth and invasion of lung adenocarcinoma cells through regulation of TGFB1/SMAD3 signaling. Int J Immunopathol Pharmacol. 2015;28(1):45‐52. 25816405 10.1177/0306419015572073

[23] Spinola M , Falvella FS , Colombo F , et al. MFSD2A is a novel lung tumor suppressor gene modulating cell cycle and matrix attachment. Mol Cancer. 2010;9:62. 20236515 10.1186/1476-4598-9-62 PMC2846890

[24] Schlegel CR , Georgiou Ml , Misterek MB , et al. DAPK2 regulates oxidative stress in cancer cells by preserving mitochondrial function. Cell Death Dis. 2015;6:e1671. 25741596 10.1038/cddis.2015.31 PMC4385915

[25] Ko JM , Chan Pl , Yau Wl , et al. Monochromosome transfer and microarray analysis identify a critical tumor‐suppressive region mapping to chromosome 13q14 and THSD1 in esophageal carcinoma. Mol Cancer Res. 2008;6(4):592‐603. 18403638 10.1158/1541-7786.MCR-07-0154

[26] Muharram G , Sahgal P , Korpela T , et al. Tensin‐4‐dependent MET stabilization is essential for survival and proliferation in carcinoma cells. Dev Cell. 2014;29(5):629‐630. 28898622 10.1016/j.devcel.2014.05.018 PMC5628947

[27] Yan Y , Jiang Y . RACK1 affects glioma cell growth and differentiation through the CNTN2‐mediated RTK/Ras/MAPK pathway. Int J Mol Med. 2016;37(1):251‐257. 26718491 10.3892/ijmm.2015.2421

[28] Zhang YL , Li Q , Yang XM , et al. SPON2 promotes M1‐like macrophage recruitment and inhibits hepatocellular carcinoma metastasis by distinct integrin‐Rho GTPase‐hippo pathways. Cancer Res. 2018;78(9):2305‐2317. 29440144 10.1158/0008-5472.CAN-17-2867

[29] Bikkavilli RK , Avasarala S , Van Scoyk M , et al. Wnt7a is a novel inducer of beta‐catenin‐independent tumor‐suppressive cellular senescence in lung cancer. Oncogene. 2015;34(42):5317‐5328. 25728679 10.1038/onc.2015.2 PMC4558401

[30] Brady JJ , Chuang CH , Greenside PG , et al. An Arntl2‐driven secretome enables lung adenocarcinoma metastatic self‐sufficiency. Cancer Cell. 2016;29(5):697‐710. 27150038 10.1016/j.ccell.2016.03.003 PMC4864124

[31] Zhan P , Zhang B , Xi GM , et al. PRC1 contributes to tumorigenesis of lung adenocarcinoma in association with the Wnt/beta‐catenin signaling pathway. Mol Cancer. 2017;16(1):108. 28646916 10.1186/s12943-017-0682-z PMC5483280

[32] Wang T , Zhang L , Tian P , Tian S . Identification of differentially‐expressed genes between early‐stage adenocarcinoma and squamous cell carcinoma lung cancer using meta‐analysis methods. Oncol Lett. 2017;13(5):3314‐3322. 28521438 10.3892/ol.2017.5838 PMC5431262

[33] Ning P , Wu Z , Hu A , et al. Integrated genomic analyses of lung squamous cell carcinoma for identification of a possible competitive endogenous RNA network by means of TCGA datasets. PeerJ. 2018;6:e4254. 29340250 10.7717/peerj.4254 PMC5768173

[34] Kwon YJ , Lee SJ , Koh JS , et al. Genome‐wide analysis of DNA methylation and the gene expression change in lung cancer. J Thorac Oncol. 2012;7(1):20‐33. 22011669 10.1097/JTO.0b013e3182307f62

[35] Ooi AT , Gower AC , Zhang KX , et al. Molecular profiling of premalignant lesions in lung squamous cell carcinomas identifies mechanisms involved in stepwise carcinogenesis. Cancer Prev Res (Phila). 2014;7(5):487‐495. 24618292 10.1158/1940-6207.CAPR-13-0372 PMC4059064

[36] Wright CM , Savarimuthu Francis SM , Tan ME , et al. MS4A1 dysregulation in asbestos‐related lung squamous cell carcinoma is due to CD20 stromal lymphocyte expression. PLoS ONE. 2012; 7(4):e34943. 22514692 10.1371/journal.pone.0034943 PMC3325913

[37] Woenckhaus M , Klein‐Hitpass L , Grepmeier U , et al. Smoking and cancer‐related gene expression in bronchial epithelium and non‐small‐cell lung cancers. J Pathol. 2006;210(2):192‐204. 16915569 10.1002/path.2039

[38] Han SS , Kim WJ , Hong Y , et al. RNA sequencing identifies novel markers of non‐small cell lung cancer. Lung Cancer. 2014;84(3):229‐235. 24751108 10.1016/j.lungcan.2014.03.018

[39] Wang Y , Mei Q , Ai YQ , et al. Identification of lung cancer oncogenes based on the mRNA expression and single nucleotide polymorphism profile data. Neoplasma. 2015;62(6):966‐973. 26458310 10.4149/neo_2015_117

